# A Case of Pyriform Sinus Fistula Infection with Double Tracts

**DOI:** 10.1155/2014/126840

**Published:** 2014-10-27

**Authors:** Masato Shino, Yoshihito Yasuoka, Kyoko Nakajima, Kazuaki Chikamatsu

**Affiliations:** Department of Otolaryngology-Head & Neck Surgery, Gunma University Graduate School of Medicine, 3-39-22 Showa-machi, Maebashi, Gunma 371-8511, Japan

## Abstract

Pyriform sinus fistula is a rare clinical entity and the precise origin remains controversial. The fistula is discovered among patients with acute suppurative thyroiditis or deep neck infection of the left side of the neck and is usually located in the left pyriform sinus. To the best of our knowledge, only a single tract has been reported to be responsible for pyriform sinus fistula infection. We present a case of a 13-year-old female patient with a pyriform sinus fistula that caused a deep infection of the left side of the neck and showed double-tract involvement discovered during surgical resection of the entire fistula. Both tracts arose around the pyriform sinus and terminated at the upper portion of the left lobe of the thyroid.

## 1. Introduction

Pyriform sinus fistula is a rare clinical entity that is incidentally found associated with acute suppurative thyroiditis or deep neck infection. This congenital internal fistula arises from the apex of the pyriform sinus of hypopharynx, penetrates the cricothyroid muscle, and terminates in or adjacent to the dorsolateral portion of the left lobe of the thyroid. Sandborn and Shafer [1] first reported a case of a branchial cleft cyst in 1972. Takai et al. [2] described seven cases of thyroiditis that were caused by bacterial infection through the pyriform sinus fistula. The fistula has been believed to be a remnant related to the third or fourth pharyngeal pouches that were present during embryonic development. Miyauchi et al. [3] stated that the fistula was a remnant related to the ultimobranchial body. However, the precise origin of the internal fistula remains controversial. Although many papers and case reports describing diagnosis, treatment, and right-sided fistula cases have been published, no reports have described double pyriform sinus fistulae. Here, we report a case of pyriform sinus fistula infection with double tracts.

## 2. Case Report

**Figure 1:**
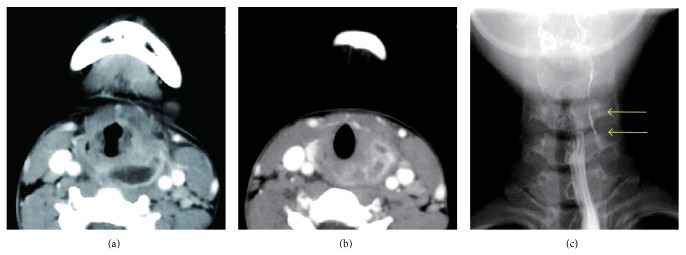
(a) A computed tomographic scan showing a deep neck infection that was located between the common carotid artery and hypopharynx. (b) The infection reached the thyroid beyond the capsule and occurred with acute suppurative thyroiditis. (c) Barium swallow examination shows obvious leakage of barium from the left pyriform sinus, which indicated the presence of a fistula (arrows). Only one fistula was identified in this test.

**Figure 2:**
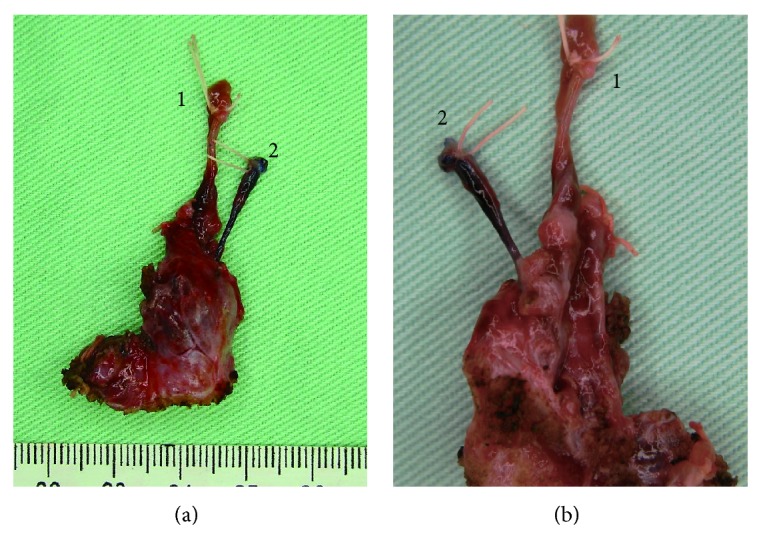
(a) Anterior view of the two resected tracts and a part of the thyroid. 1 shows a brownish tract that terminated at the upper part of the thyroid. 2 showed a crystal violet-stained tract that ended lower than the brownish tract and on the lateral side of the thyroid compared with 1 tract. (b) The posterior view of the resected tissue. The crystal violet-stained tract 2 was attached lower and more lateral than the brownish tract 1.

A 13-year-old female was brought to our hospital because of swelling of the left side of the neck. Several years earlier, a similar episode occurred, and administration of intravenous antibiotics improved the patient's symptoms. The left side of the neck was swollen, and her skin was slightly reddish. The patient felt pain in her neck and could not drink and eat; however, there was no dyspnea. The body temperature was 37.8°C. A blood test showed an increase of white blood cells and a high level of C-reactive protein. Her thyroid hormone levels were also increased, but thyroid stimulating hormone was decreased, which indicated acute suppurative thyroiditis. Enhanced computed tomography was performed on the same day and showed that the abscess was located between the common carotid artery and hypopharynx. The abscess extended from the height of the hyoid bone to the posterior of the left lobe of the thyroid. The inflammation partially reached beyond the thyroid capsule (Figures 1(a) and 1(b)). Therefore, we performed a drainage operation under general anesthesia. The abscess cavity was located deeper than the sternocleidomastoid muscle and between the common carotid artery and hypopharynx. The strap muscles, sternocleidomastoid muscle, inferior pharyngeal constrictor muscle, and part of the thyroid were affected by inflammation. The pus was discharged that had partially reached the buccopharyngeal space. No communication with the pharyngeal space and abscess cavity was observed. After the drainage operation, the postoperative course was good, and the patient was discharged. One month after the drainage operation, a barium swallow study revealed that a 3 cm fistula located at the apex of the left pyriform sinus extending inferiorly (Figure 1(c), yellow arrows). No other tracts were identified. Therefore, we diagnosed this case as left pyriform sinus fistula infection resulting in a deep neck infection and acute suppurative thyroiditis. Recently, an endoscopic chemocauterization technique has been described [4–7]. However, because we consider that operative excision of the entire tract, including the adjacent thyroid tissue, is essential to prevent recurrence, we performed a surgery using a lateral cervical approach. First, a direct laryngomicroscope was inserted orally to identify an internal opening of the left pyriform sinus tract. The opening was identified at the posterior of the fold of the internal branch of the superior laryngeal nerve at the left side of the pyriform sinus, and crystal violet was injected into the opening of the tract. There was only one internal opening. Next, a skin incision was made along the scar of the previous surgery. The subcutaneous tissue and muscles were carefully divided to detect the violet-stained tract. In the front of the area between the cricoid cartilage and inferior horn of the thyroid cartilage, a dark brownish tract was observed that arose from the inside of the inferior pharyngeal constrictor muscle. The tract reached the left lobe of the thyroid and invaded the capsule of the thyroid. However, continuity of the brownish tract with the left pyriform sinus was not confirmed. Therefore, the brownish tract was ligated as tightly as possible to the left pyriform sinus. Because the tract was not stained by crystal violet, we further divided the muscles to detect the crystal violet-stained tract. Subsequently, an intended tract, that is, the crystal violet-stained tract, was found. The tract originated from the left pyriform sinus and ended at the adjacent upper portion of the left side of the thyroid. This tract was also ligated at the outlet of the left pyriform sinus. The two tracts were resected together with a portion of the left lobe of the thyroid. The brown tract was terminated superior to the crystal violet-stained tract (Figure 2).

## 3. Pathological Findings

**Figure 3:**
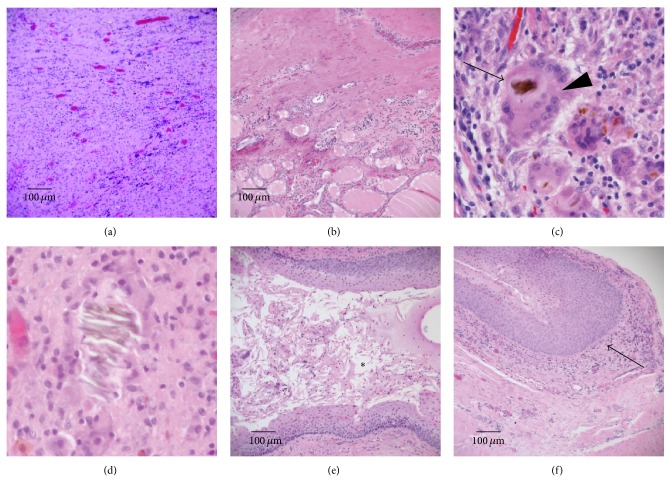
Pathological findings by hematoxylin and eosin staining of the brownish tract ((a)–(d)) and the crystal violet-stained tract ((e), (f)) and a view of the ends of the tracts ((b), (f)). (a) Epithelium of the lumen was completely replaced by granulation tissue and inflammatory cells. (b) The end of the brownish tract has invaded the thyroid beyond the capsule. (c) The brownish tract contained a foreign substance (arrow) that was surrounded by multinucleated giant cell (arrowhead). (d) Another foreign substance was seen in the granulation tissue of the brownish tract. These substances are observed everywhere, which proved the previous communication with the pharyngeal lumen. (e) An internal lumen (asterisk) covered by epithelium has obviously formed. (f) The terminus is a blind end (arrow) that does not enter the thyroid across the capsule.

The brownish tract (1 in Figures 2(a) and 2(b)) had not formed an open lumen. The epithelium was completely replaced by granulation tissue (Figure 3(a)). The muscular tissue was not seen either. At the caudal side, the brownish tract had entered the inside of thyroid across the capsule (Figure 3(b)). The granulation tissue of the brownish tract contained a foreign substance (Figure 3(c) arrow) that was surrounded by multinucleated giant cell (Figure 3(c) arrowhead). Other foreign substances like Figure 3(d) were observed here and there. The existence of these foreign substances was suspected to have enabled communication with the pharyngeal lumen. In the violet-stained tract (2 in Figures 2(a) and 2(b)), an internal lumen (Figure 3(e) asterisk) covered by the epithelium was obviously present. On the thyroid side, the tract had a blind end and did not penetrate the capsule of the thyroid (Figure 3(f) arrow). This tract also contained a foreign substance in the lumen. In both tracts, C-cells were not observed by immunostaining and residual thymus-derived tissue was not found. These two tracts ran independently and terminated at different area of the upper portion of the thyroid (Figures 3(b) and 3(f)). There was no continuity between the two tracts.

## 4. Discussion

We experienced a case of pyriform sinus fistula infection with double tracts. To the best of our knowledge, there have been no other reported cases of two or more tracts contributing to pyriform sinus fistula infection. In the present case, the two tracts ran independently and showed different pathological findings.

The brownish tract originated from inside of the inferior pharyngeal constrictor muscle and ran across the front of the cricoid muscle and into the thyroid. The lumen was filled with granulation tissue pathologically, which led to speculation that severe inflammation had occurred and the normal epithelium had been completely replaced as described in a previous paper [1]. The caudal side of the brownish tract pathologically entered the inside of thyroid across the capsule. The fact that foreign substances were found in the granulation tissue of the tract suggested that the brownish tract had communication with the oral or pharyngeal space. In contrast, the crystal violet-stained tract clearly arose from the left pyriform sinus, penetrated the muscles, ran between the cricoid cartilage and the inferior horn of the thyroid cartilage, and terminated near the upper portion of the left lobe of the thyroid. The pathway was considered to be a typical fistula derived from the ultimobranchial body, a finding similar to one previously reported [3, 8]. The tract also formed the internal lumen covered with the epithelium for full length in a pathological finding. In addition, the existence of the foreign bodies proved that there had been a communication with the oral or pharyngeal space. Therefore, we concluded that a double-tract fistula existed in this patient on the basis of the reasons mentioned above. There was the possibility that the brownish tract may have been an iatrogenic scar resulting from the previous drainage operation. However, it was difficult to regard that repeated severe infections occurred due to inflammation of the crystal violet-stained tract, because of the intact lumen of the tract for whole length and no invasion of its blind end into the thyroid. Accordingly, it was highly likely that the brownish tract was not formed iatrogenically. No existence of residual muscular tissues in the brownish tract also supported our presumption. In the present case, these findings suggested that the brownish granulation replaced tract was responsible for the left side deep neck infection. Furthermore, the crystal violet-stained tract did not invade the thyroid, whereas the brownish tract led into the thyroid, which accounts for the presence of the acute suppurative thyroiditis. A possible explanation for why the internal opening of the brownish tract at the left pyriform sinus could not be identified and why a barium swallow study revealed only one fistula is that repeated severe infections of the fistula resulted in complete obstruction of the internal opening and the lumen of the tract and replacement of the epithelium with granulation tissue, consistent with a previous literature report describing a case involving obliteration of the epithelium following an acute infection [1].

Pyriform sinus fistula is a congenital anomaly that arises from disturbances in the development of the fetal branchial apparatus. There are several hypotheses regarding the specific origin of these fistulae. Miyauchi et al. [3] pathologically demonstrated that the ultimobranchial body, vestige of fifth pharyngeal pouch rather than the fourth pharyngeal pouch, was the true origin of the fistula because the parathyroid gland derived from the fourth pharyngeal pouch was not connected with the tract, although the end of the tract was close. In our case, the violet-stained tract had an internal opening at the posterior of the fold of the internal branch of the superior laryngeal nerve, penetrated the inferior pharyngeal constrictor muscle, ran between the cricoid cartilage and the inferior horn of thyroid cartilage, and terminated in an area adjacent to the upper portion of the left lobe of the thyroid. This pathway corresponded to the remnants of the ultimobranchial body, although calcitonin-producing C-cells were not present in this case. As mentioned above, the violet-stained tract was derived from the ultimobranchial body that might be remnant from the fifth pharyngeal pouch. Meanwhile, the origin of the brownish granulation replaced tract was controversial. An opening at the left pyriform sinus was not found; in contrast, the ending in the upper region of the thyroid was obvious and was superior to that of the crystal violet-stained tract (Figures 2(a) and 2(b)). The crystal violet-stained tract was derived from the ultimobranchial body [9]. The facts that the ending of the brownish tract was superior to that of the crystal violet-stained tract and the pathway of the former was ventral to that of the latter speculated that a possible candidate of the origin of the brownish tract was the fourth pharyngeal pouch. However, there is no consensus opinion on the precise origin of pyriform sinus fistula, and further study is required.

## 5. Conclusion

We first reported a case of a pyriform sinus fistula infection with double tracts. One was a crystal violet-stained tract and the other was granulation replaced tract without internal lumen structure due to repeated infections.
